# Construction of 3D lithium metal anode using bi-functional composite separator: a new approach for lithium battery†

**DOI:** 10.1039/d3ra06129a

**Published:** 2023-10-13

**Authors:** Fengquan Liu, Tianqi Xiang, Jinxin Xue, Sixin Jia, Jun Yan, Hong Huo, Jianjun Zhou, Lin Li

**Affiliations:** a College of Textiles & Clothing, State Key Laboratory of Bio-Fibers and Eco-Textiles, Qingdao University Qingdao 266071 China; b Beijing Key Laboratory of Energy Conversion and Storage Materials, College of Chemistry, Beijing Normal University Beijing 100875 P.R. China pla_zjj@bnu.edu.cn lilinll@bnu.edu.cn

## Abstract

With the increasing use of Li batteries for storage, their safety issues and energy densities are attracting considerable attention. The Li metal battery (LMB) with limited capacity in the Li metal anode is one of ideal high energy-density systems due to eliminating the use of traditional anode, elevating the energy density of battery and reducing production costs. However, the side reactions between the electrolyte and metallic Li and the irreversible loss of lithium resources caused by the generation of “dead Li” will directly lead to the loss of battery capacity during the cycling process. Therefore, the cycle life of the LMB with limited capacity in the Li metal anode faces significant challenges. Herein, a bi-functional manganese oxide (MnO)/polypropylene/Li_1+*x*_Al_*x*_Ti_2−*x*_(PO_4_)_3_ (LATP) composite separator is designed to construct a stable three dimensional (3D) Li metal in the surface of Cu foil for LMB. The MnO can dissolve in electrolytes with low concentration, which can be reduced to produce Mn and Li_2_O, functioning as nucleating seeds to induce sheet-like Li deposition. The sustainably released MnO also involves in the formation of solid electrolyte interphase (SEI) layer, which can be repaired promptly once damaged by the volume expansion of Li. The LATP coating layer is *in situ* transferred onto the sheet-like Li, acting as an artificial SEI layer for further protection. The constructed 3D Li metal anode with limited capacity shows improved cycle stability in LiFePO_4_ cell, which shows a capacity retention of 94.5% after 150 cycles. Our strategy, constructing stable 3D Li metal anode with bi-functional composite separator, will bring a new inspiration for developing high energy density LMB.

## Abstract 2

A feasible strategy of constructing stable 3D Li metal anode with bi-functional composite separator provides a new idea for developing high energy density lithium metal batteries.

## Graphical abstract

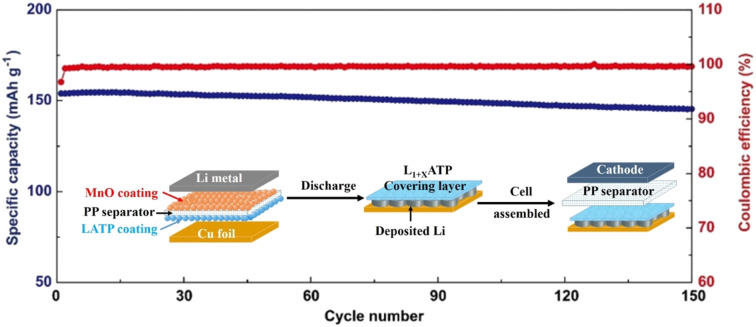

## Introduction

With the continuous development of science and technology, secondary batteries with higher energy-density are urgently needed for both electric vehicles and mobile electronics.^1–3^ Lithium (Li) metal possesses the highest theoretic capacity (3860 mA h g^−1^) and the lowest standard reduction potential (−3.04 V *vs.* standard hydrogen electrode), and Li metal batteries (LMBs) are perspective high energy-density batteries for future energy storage devices.^4,5^ Commonly prepared LMBs usually use commercial Li metal foil with the thickness of 300–500 μm (60–100 mA h cm^−2^), which has a capacity much higher than that of coupled cathode (4–5 mA h cm^−2^, corresponding to Li foil thickness of 20–25 μm).^6^ Too much excessive amounts of Li will bring great safety risk since metallic Li has high reactive activity. Furthermore, dendritic deposited Li might penetrate the separator and lead to short-circuit, fire or explosion. When excessive Li is used as an anode, volume expansion/shrink in the Li metal anode will inevitably occur during charging/discharging cycles, which brings great challenges for stable cycle performance.^7,8^ Excessive Li is also detrimental to achieving high energy-density for LMBs. As a result, there should be a limit for the capacity of Li metal anodes. However, LMB with limited capacity in Li metal anode often shows unsatisfactory cycle performance.^9^ Various strategies have been taken to modify the Li metal anodes for improving the electrochemical performance of LMBs.

A strategy is to change the composition and species of liquid electrolytes (LEs). It has been reported that high concentration Li salts,^10,11^ all-fluorinated ether electrolytes,^12^ weakly-solvating electrolyte,^13^ and electrolyte additives (LiNO_3_,^14^ vinylene carbonate,^15^ fluoroethylene carbonate,^16^*etc.*) could promote the uniform deposition of Li metal and improve the cycle performance of LMBs. However, Li salts of high concentration endow the LE with high viscosity, which is adverse to its permeation into the electrodes. Construction of artificial solid electrolyte interface (SEI) membrane is also an effective way to improve its interfacial stability.^17,18^ Some polymer or inorganic substances (such as poly(dimethylsiloxane),^19^ hexagonal boron nitride,^20^ TiO_2_,^21^ sulfonated poly(vinyl alcohol),^22^*etc.*) were often coated on the surface of Li metal anodes to prevent LE corrosion. Modifying separator, as an efficient strategy, not only protects Li anodes, but also is easy to achieve batch preparation and have strong repeatability. Functional coating layers such as MnCO_3_,^9^ g-C_3_N_4_,^23^ or PbZr_0.52_Ti_0.48_O_3_ ^24^ were reported to *in situ* transfer from the separator and act as an artificial SEI layer to protect Li metal anode surface. Recently, our group reported that composite separators with transition metal oxides as a coating layer could modify the dendrite-free Li deposition by sustainably releasing metal ions as nucleating agents.^25^ However, the spontaneous reaction between functional coating layer and metallic Li will consume a large amount of active material Li, resulting in irreversible loss of capacity for battery. Consequently, constructing stable Li metal anode with limited capacity is still a big challenge.

**Fig. 1:**
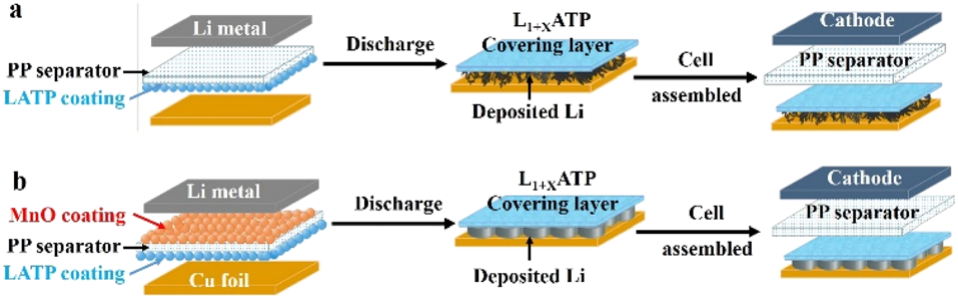
Schematic diagrams of 3D lithium metal anodes based on different composite separators (a) PP/LATP and (b) MnO/PP/LATP.

In this paper, a bi-functional MnO/polypropylene (PP)/Li_1+*x*_Al_*x*_Ti_2−*x*_(PO_4_)_3_ (LATP) composite separator was prepared, and a kind of 3D Li metal anode on copper foil with good electrochemical performance was obtained by using Li‖Cu cells. LATP was one of NASICON-type solid electrolytes, a good Li^+^ conductor stable in the atmosphere and feasible to be coated on the surface of separator outside of the glove box.^26,27^ LATP could react with Li metal spontaneously, which could be *in situ* transferred from the composite separator to Li metal surface and form an artificial SEI layer as shown in Fig. 1a. MnO is used to promote the formation of dendrite-free deposition with a stable SEI layer.^25^ Based on the LATP/PP/MnO bi-functional composite separator, a 3D Li metal anode was constructed as shown in Fig. 1b. The 3D Li metal anodes could be coupled with LiFePO_4_ (LFP) cathodes, and the cells could run well for more than 150 cycles with the capacity retention of 94.5%, promoting the development the further AF-LMBs.

## Experimental section

### Preparation of composite separators

The LATP/PP composite separators were prepared by coating LATP slurry on one side of bare PP separator. LATP slurry was prepared by high-energy ball-milling the mixture of nanoscale LATP particles (22.5 wt%) with polyethylene oxide (PEO) (2.5 wt%) in *N*-methylpyrrolidone (NMP) (75.0 wt%), which was uniformly coated on the PP separator (20.0 μm, Cangzhou Mingzhu Plastic Co., Ltd, China) with the thickness of ∼3.5 μm. The composite separator was dried at 60 °C for 10 hours before using.

The LATP/PP/MnO composite separators were prepared by coating MnO slurry on the other side of LATP/PP separator. MnO slurry was prepared by high-energy ball-milling the mixture of MnO powder (40.0 wt%) with sodium polyacrylate (0.5 wt%, dispersing agent), a wetting agent (1.0 wt%), a polyacrylate binder (2.0 wt%) and a carboxymethyl cellulose thickener (2.0 wt%) in deionized water (54.5 wt%). The thickness of MnO coating layer was ∼3.5 μm. The composite separator was dried at 60 °C for 10 hours before using. The gravimetric loading of LATP and MnO in MnO/PP/LATP separator is about 0.5 and 1.0 mg cm^−2^, respectively. The properties of the separator were showed in Table S1.†

### Characterization methods

The morphologies and element mapping images were measured by scanning electron microscopy (SEM, SU8000, Hitachi) equipped with an energy dispersive X-ray spectroscopy (EDS, XFlash6160, Bruker). All samples were washed many times with 1,2-dimethoxyethane (DME, Sigma-Aldrich) solvent to remove residual electrolyte before tested.

The powder X-ray diffraction (XRD) data of pristine LATP and reduced LATP were obtained by a Phillips X'pert Pro MPD diffractometer with Cu-Kα (*λ* = 0.15418 nm). LATP powders were directly dropped on Li metal foil, and certain organic solvent was added to promote the reduction reaction between Li and LATP. After sufficient reaction, the reduced LATP powders was characterized with XRD.

The element valence states of the pristine LATP and reduced LATP were characterized by X-ray photoelectron spectroscopy (XPS, VG Scientific Ltd, UK). The sample preparation method of reduced LATP was the same as that of XRD sample. The spectra were calibrated by C1s (284.8 eV) to eliminate surface charge effects.

### Coin cells assembly and electrochemical characterization

The electrolyte was 1.0 mol L^−1^ (M) bis(trifluoromethane) sulfonamide lithium salt (LiTFSI, Sigma-Aldrich) in a mixed solvent of DME and 1,3-dioxolane (DOL) (v/v = 1 : 1) with 1.0 wt% LiNO_3_ as additive (Shenzhen Biyuan Electronics Co., Ltd, China).

The Li‖Cu cells were assembled with different separators (PP/LATP or MnO/PP/LATP separator). When the cells were assembled, the LATP coating layer of the composite separator contacted the Cu foil. After plating various capacities of Li, the cells were disassembled to obtain 3D Li metal anodes.

LFP cathode slurry was prepared by mixing LFP, conductive carbon and the polyvinylidene fluoride (PVDF) binder (weight ratio = 85 : 10 : 5) in NMP. The slurry was spread on the aluminum (Al) foil to obtain LFP cathode. 3D Li‖LiFePO_4_ cells were assembled to evaluate the performance of the 3D Li metal anodes. The area loading of LFP cathode is about 9.6 mg cm^−2^, corresponding to 1.48 mA h g^−1^, respectively. The LFP cells are cycled at 2.5–4.0 V on a CT2001A battery testing system.

The NCM cathode was prepared with same procedure as that of LFP cathode by using NCM622. The mass loading of NCM622 is about 9.6 mg cm^−2^. CR2032 coin cells were assembled with 3D Li as anodes in an Ar-filled glove box with the same amount of electrolyte (70.0 μL). The NCM622 cells are cycled at 2.8–4.3 V. The cyclic voltammetry (CV) was collected *via* an electrochemical workstation (Interface 1000E, Gamry Instruments). Electrochemical impedance spectroscopy (EIS) was tested by an electrochemical working station (Interface 1000E, Gamry Instruments) over a frequency range of 10^6^–0.1 Hz with a disturbance amplitude of 5 mV.

## Results and discussion

**Fig. 2:**
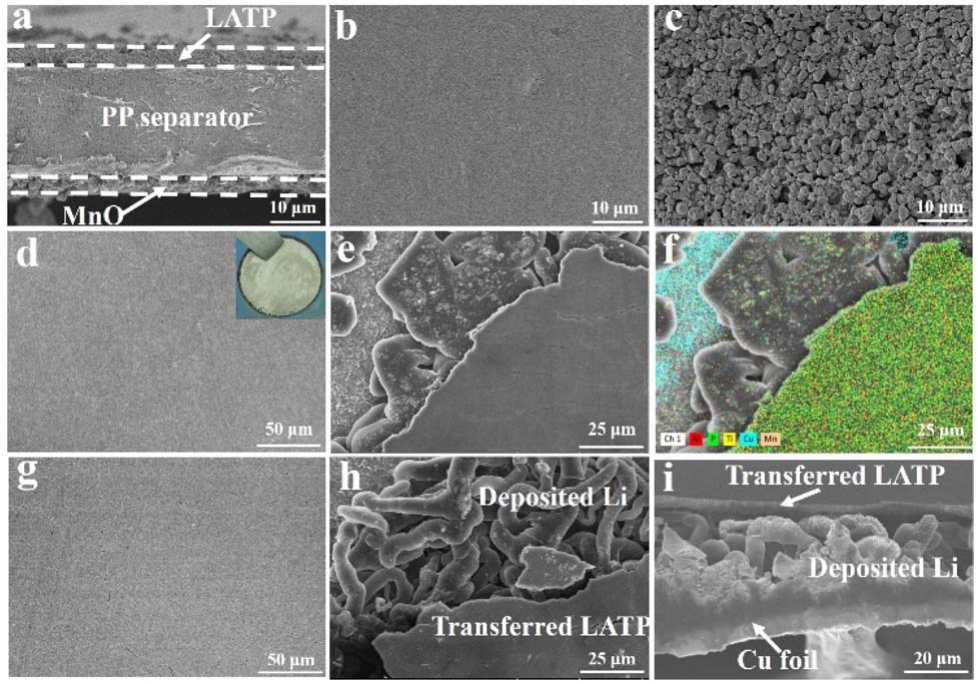
(a) The cross-sectional SEM image of LATP/PP/MnO composite separator. The top view SEM images of (b) LATP coating and (c) MnO coating, respectively. (d) Top view SEM image of covering layer of 3D Li metal anode. (e and f) SEM image and its element mapping for 3D Li metal anode obtained by Li|MnO/PP/LATP|Cu cell at 0.5 mA cm^−2^ for 4.0 mA h cm^−2^ in 1.0 M LiTFSI electrolyte (DOL : DME = 1 : 1, 1 wt% LiNO_3_). Inset of (d) was the photo of 3D Li metal anode. (g) The cross-sectional and (h and i) top view SEM images of covering layer of 3D Li metal anode with different magnification obtained by Li|PP/LATP|Cu cell at 0.5 mA cm^−2^ for 4.0 mA h cm^−2^ in 1.0 mol L^−1^ LiTFSI electrolyte (DOL : DME = 1 : 1, 1 wt% LiNO_3_).

The bi-functional MnO/PP/LATP composite separator was prepared *via* a simple coating process in atmospheric environment. As shown in Fig. 2a, the thickness of PP separator is about 20 μm, and both the thicknesses of LATP and MnO coating layers are ∼3.5 μm, respectively. The LATP coating layer is a dense layer with PEO as a binder (Fig. 2b), while the MnO coating layer has a porous structure (Fig. 2c). The 3D Li metal anodes were formed in Li‖Cu cells using the MnO/PP/LATP composite separators with LATP layer facing the Cu foils. When the Li‖Cu cells were discharged, Li was deposited on the Cu foil. Owing to the strong reducibility, the freshly deposited Li can react with almost all kinds of materials, including LATP. Ti^4+^ in LATP can be reduced to Ti^3+^ with the following reaction equation:^27,28^*y*Li + Li_1+*x*_Al_*x*_Ti_2−*x*_(PO_4_)_3_ → Li_1+*x*+*y*_Al_*x*_Ti_2−*x*_(PO_4_)_3_

The deposited Li on Cu foil is chemically bonded with the LATP coating layer due to this reaction. When the Li‖Cu cells were disassembled, it could be observed that the LATP layer had been *in situ* transferred onto the Cu current collector, covering the surface of deposited Li and forming 3D Li metal anode (Fig. 2d–f). Top view SEM image of the covering layer shows flat surface without obvious crack (Fig. 2d). The optical image also shows that the surface of deposited Li on Cu foil is completely covered by LATP layer with pale yellow (inset of Fig. 2d). From edge of the obtained 3D Li metal anodes, tri-layer structure can be observed as shown in Fig. 2e and f. The dense layer on the top is rich in P, Ti and Al elements, verifying the transferred LATP layer. Cu element can be observed on the bottom, indicative of the copper current collector. The sheet-like parts in the middle layer that can't be discriminated in element mapping are the deposited Li, which has low tortuosity and small specific surface area.

The SEM images indicate that 3D Li metal anode on the copper foil has been constructed with sheet-like deposited Li covered by a dense LATP layer on the top. The dense LATP covering layer can act as an artificial SEI layer, since LATP, reduced LATP and PEO are conductors for Li^+^, which is beneficial for preventing the deposited Li from corroded by LE. PEO was selected as a binder due to the appropriate adhesive force to the PP separator, which can contribute to the completely transferring of the LATP layer. If PVDF with higher adhesive force was chosen as a binder for LATP coating, only part of the LATP layer could be *in situ* transferred, and small patches were randomly observed on the deposited Li surface (Fig. S1 and S2†). The MnO coating layer is effective in modifying Li deposition with reduced specific surface area, which has been proved previously.^25^ Furthermore, the MnO coating layer can endow the SEI film with more inorganic components and better stability since the slightly dissolved MnO can be reduced to form metallic Mn and Li_2_O on the broken sites of the SEI film.^25^ Though the MnO layer shows obvious effect in modifying Li deposition and improving the stability of the SEI film, it consumes too much Li capacity when contacting with Li foil or freshly deposited Li. So the MnO layer didn't directly contact with the freshly deposited Li during preparing the 3D Li metal anode. If no MnO layer was used on the PP/LATP composite separator when preparing 3D Li metal anode in the Li‖Cu cells, the flat transferred LATP covering layer can still be observed (Fig. 2g). However, the deposited Li beneath the transferred LATP layer on Cu foil is dendritic (Fig. 2h and i), which further confirms the role of MnO coating layer in modifying Li deposition.

**Fig. 3:**
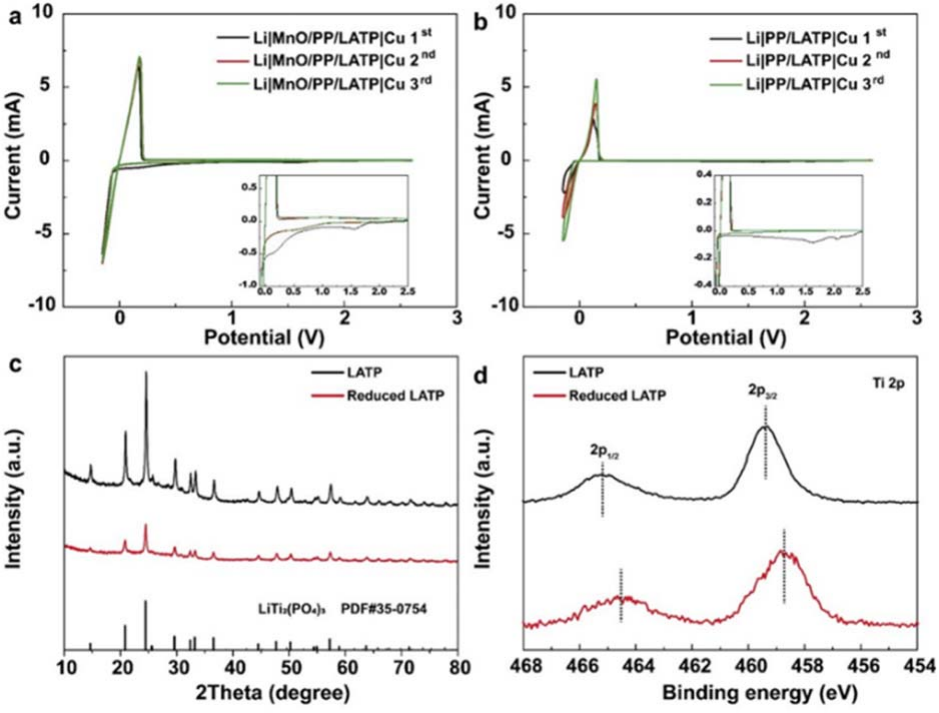
(a and b) CV curves of Li‖Cu cells with different separators scanning from OCV to −0.15 V at the rate of 0.15 mV s^−1^ in 1.0 M LiTFSI electrolyte (DOL : DME = 1 : 1). (c) XRD and (d) XPS profiles of pristine LATP and reduced LATP, respectively.

CV, XRD and XPS tests were performed to explore the mechanism of the composite separators. CV curves of Li‖Cu cells with different separators were scanned from open circuit voltage (OCV) to −0.15 V and then back to OCV as demonstrated in Fig. 3a and b. In the first cathodic cycle, the peaks beginning at ∼2.3 and 2.06 V can be attributed to the reduction of LE.^29^ The peaks at ∼1.6 V can be attributed to the reduction of LATP, which disappears in the first anodic cycle, suggesting the irreversible Li consumption by reaction with LATP coating layer.^25^ Another peak beginning at about 0.7 V in Fig. 3a can be attributed to the reduction of MnO, which consistently appears in the second and third cycles implying the continuous impact of MnO on Li deposition. The Li plating/stripping current is much larger with smaller fluctuation in the Li‖Cu cells with MnO/PP/LATP composite separator, indicating that the transferred LATP layer with sheet-like Li deposition has endowed a stable SEI layer on the electrode with better dynamic response (Fig. 3a).

The XRD profiles of pristine and reduced LATP were showed in Fig. 3c, which are consistent with the profile of LiTi_2_(PO_4_)_3_ (PDF # 35-0754). The reduced LATP is in an amorphous state,^27^ and the observed weaker peak intensity in the XRD profiles suggests that only part of LATP has been reduced (Fig. 3c). XPS spectra were given to indicate the element valence state (Fig. 3d). The XPS peaks of Ti 2p in reduced LATP are shifted to lower binding energy relative to pristine LATP, which can be attributed to the reduction of Ti^4+^ to Ti^3+^.

3D Li metal anodes with the capacity of 4.00 mA h cm^−2^ was deposited on Cu current collector with mono-functional PP/LATP and bi-functional MnO/PP/LATP separators. The effective capacities of the 3D lithium anodes were 3.88 and 3.83 mA h cm^−2^, calculated from the Coulombic efficiencies (CEs) of 96.89% and 95.76%, respectively (Fig. S3†). As previously stated, the 3D Li metal anode has a tri-layer structure, consisting of Cu foil, deposited Li and a reduced LATP covering layer. The difference of two 3D Li metal anodes exists in the Li deposition morphology, *i.e.* coarse Li dendrites with mono-functional PP/LATP separator (name as mono-protected Li) (Fig. 2g–i) and sheet-like Li with bi-functional MnO/PP/LATP separator (named as bi-protected Li) (Fig. 2d–f).

**Fig. 4:**
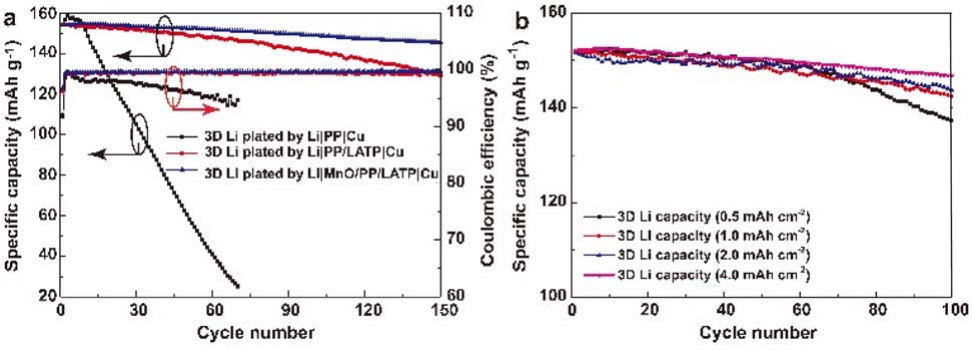
(a) Cycle performance of 3D Li‖LFP cells (N/P = 2.70) with PP separators in 1.0 M LiTFSI electrolyte (DOL : DME = 1 : 1, 1.0 wt% LiNO_3_) at 0.5C. The 3D Li with the capacity of 4.0 mA h cm^−2^ was obtained by using Li‖Cu cells with different separators in 1.0 M LiTFSI electrolyte (DOL : DME = 1 : 1, 1.0 wt% LiNO_3_). (b) Cycle performance of 3D Li‖LFP cells with the 3D Li anodes of different areal capacities plated in Li‖Cu cells with MnO/PP/LATP composite separator.

LMBs were assembled to evaluate the battery performance of the 3D Li metal anodes in ether-based electrolyte (Fig. 4a). Three kinds of coin cells have almost equal initial capacity of about 154.0 mA h g^−1^. The cell with PP separator exhibits poor cycling performance and low CE. The discharge capacity is 25.1 mA h g^−1^ after 70 cycles, corresponding to a capacity retention ratio of 16.2%. The CE decreases with cycle numbers, which indicates that the SEI is extremely unstable and a large amount of the active materials have lost at every cycle. The average CE of the cell with mono-protected Li anode is 99.18%, lower than that of the cell with bi-protected Li (99.24%), suggesting less stability of the SEI layer and more side reactions with LE. Considering the Li deposition structure in the mono-protected Li anode, lower CE seems reasonable for as much as LE will react more seriously with dendritic Li than sheet-like Li due to much larger specific surface area. The charge/discharge profiles indicate that the polarization gradually increases in the cells with mono-protected Li anode and keeps stable in the cells with bi-protected Li anode (Fig. S4†), which further verifies the role of controlling the Li deposition morphology and stabilizing the interface of the 3D Li metal anodes. After 150 cycles, the capacities are 128.8 and 145.4 mA h g^−1^ with capacity retention rate of 83.5% and 94.5%, respectively. The cell with bi-protected Li anode decays at 0.057 mA h g^−1^ per cycle, lower than that with mono-protected Li anode (0.17 mA h g^−1^ per cycle), exhibiting better cycle stability. If Li metal anode is prepared in a Li|MnO/PP|Cu cell without the LATP transferred covering layer, the sheet-like Li‖LFP cells can only stably run for ∼120 cycles,^23^ demonstrating the advantage of the 3D Li metal anode prepared with bi-functional MnO/PP/LATP separators. EIS of the cells were showed in Fig. S5.† Before cycling, the cells with bi-protected Li anode and mono-protected Li anode have almost the same internal resistance. After 150 cycles, the internal resistance of the cells with bi-protected Li anode (≈86 Ω) is much lower than that of the cells with mono-protected Li anode (≈162 Ω) (Fig. S5b†). Since the LFP cathode is very stable, the internal resistance increase can be attributed to the reaction of active Li with liquid electrolyte on the anode, which results in the accumulation of SEI layer and “dead Li”. Less internal resistance increase is observed in cell with bi-protected Li anode, which further confirm that the constructed bi-protected 3D Li anode possesses an extraordinary interfacial stability.

Cyclic voltammetry (CV) is scanned to give further insight into the polarization in the cells. The cathodic peak appears at higher voltage in the LFP cell using the 3D Li plated by MnO/PP/LATP|Cu than that by PP/LATP|Cu. While the anodic peak appears at lower voltage with much narrower width, which means that smaller overpotential is needed for the reaction. It can also be observed that the polarization between the cathodic and anodic peaks is much less than that using mono-protected Li anode, indicating much smaller polarization in the cells with bi-protected Li anode, which is consistent with the results descripted in Fig. 4a and S5.†

In LMBs, the negative/positive (N/P) ratio affects not only the energy density but also the cycle stability. Thus, 3D Li metal anodes with other capacities were prepared with bi-functional MnO/PP/LATP separator in Li‖Cu cells, which were further evaluated with LFP cells. Fig. 4b exhibits the cycle performance of 3D Li‖LFP cells with various N/P ratio. As the N/P ratio is 0.34 (deposited Li capacity of 0.50 mA h cm^−2^), the cell can stably run for about 60 cycles. Then the cell decays in a rapid speed, indicating the possible exhaustion of plated Li. As N/P is larger than 0.68 (deposited Li capacity of 1.00 mA h cm^−2^), the cells can stably run for more than 100 cycles. When N/P is 2.70 (deposited Li capacity of 4.00 mA h cm^−2^), the cell can stably run for more than 150 cycles, which is a high performance compared with the cells using Li-free or ultra-thin Li metal anodes in the literature (Table S2†).^9,10,19,25,30–33^

**Fig. 5:**
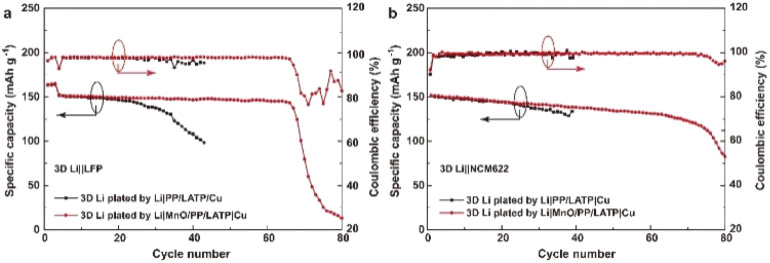
Cycling performance of (a) 3D Li‖LFP and (b) 3D Li‖NCM cells with PP separator in 1.0 M LiPF_6_ electrolyte (DMC : EC : DEC = 1 : 1 : 1, 2 wt% vinylene carbonate (VC)) at 0.1C for the first three cycles and 0.5C for the later cycles. The 3D Li anode with the capacity of 4.0 mA h cm^−2^ was obtained by Li‖Cu cells with LATP/PP/MnO composite separator in 1.0 M LiTFSI electrolyte (DOL : DME = 1 : 1, 1 wt% LiNO_3_).

Since carbonate-based LEs have wider electrochemical stability window than that of ether-based LEs, the 3D Li metal anode is further evaluated in the 3D Li‖LFP (N/P = 2.70) filled with a carbonate-based LE. As shown in Fig. 5a, both kinds of cells have similar initial capacity of about 152.0 mA h g^−1^. Compared with ether-based LE, the carbonate-based LE is more corrosive to deposited Li,^34–37^ so the cycle life is much shorter than that with ether-based LE. The cell with mono-protected 3D Li anode can only stably run for about 20 cycles. After that, the discharge capacity decays very rapidly, indicating the exhaustion of Li in a very short period. While the cell with bi-protected Li anode can stably run for about 65 cycles with a capacity retention ratio of 95.4%. 3D Li‖LiNi_0.6_Co_0.2_Mn_0.2_O_2_ (NCM622) cells (N/P = 2.70) was also assembled and filled with a carbonate-based LE. The cell with bi-protected 3D Li anode also shows much superior performance than that with mono-protected 3D Li anode (Fig. 5b). Hence, our strategy of constructing 3D Li metal anodes bi-functional composite separator is effective, which can alleviate the corrosion of LE on Li metal anode and prolong the cycle life of LMBs.

Postmortem examination was carried to investigate the performance deterioration of the 3D Li in NCM622 cell with carbonate-based electrolytes after 80 cycles. As shown in Fig. S7a,† the original sheet-like Li in Fig. 2e has become nodule-like Li with large grain size in most areas. Furthermore, pits can be observed on the surface leading to much larger specific surface area, which verifies that carbonate-based LE is more corrosive to deposited Li.^38,39^ Occasionally, deposited Li with the overgrowth of whiskers is seen on the surface instead of beneath the reduced LATP (Fig. S7b†). Yang *et al.*^27^ has reported that the electronic conductivity of LATP will increase when reduced by metallic Li. The electronic conductivity of reduced LATP is determined to be ∼2.5 × 10^−6^ S cm^−1^, much larger than that of LATP (∼5.1 × 10^−9^ S cm^−1^) (Fig. S8†). It's the increased electronic conductivity of reduced LATP that has resulted in Li depositing directly on its surface. Without the 3D structure, the deposited Li direct exposes to LE, leading to the fast exhaustion of Li and capacity decay. The results demonstrate that when inorganic solid electrolyte is used as an artificial SEI layer, the electronic conductivity increase induced by Li reduction shouldn't be neglected for long-term cycle stability.

## Conclusions

In conclusion, a bi-functional MnO/PP/LATP composite separator was prepared and used to construct 3D Li metal anode. The MnO can dissolve in electrolyte and function as nucleating seeds to modify the deposition of sheet-like Li. The LATP coating layer could be *in situ* transferred from the surface of PP separator based on the redox reaction between LATP and Li metal, forming an artificial SEI covering layer on the sheet-like Li. The covering layer could effectively suppress Li corroded by LE and contribute to stabilizing the SEI layer. LFP cells with the bi-protected Li metal anode could stably run for more than 150 cycles with the N/P of 2.70. The bi-functional composite separator can be easily produced massively using coating lines. Our strategy provides a feasible way to construct stable 3D Li metal anodes for high energy-density LMBs.

## Conflicts of interest

There are no conflicts of interest to declare.

## Supplementary Material

RA-013-D3RA06129A-s001
