# Supplementary material for: Construction of 3D lithium metal anode using bi-functional composite separator: a new approach for lithium battery
Source: RSC Adv. 2023 Oct 13;13(43):30086–91. doi: 10.1039/d3ra06129a (PMC10571094; doi:10.1039/d3ra06129a)
Supplement: RA-013-D3RA06129A-s001 [file RA-013-D3RA06129A-s001.pdf]

Electronic Supplementary Material (ESI) for RSC Advances.
This journal is © The Royal Society of Chemistry 2023

Supplementary Information

Construction of 3D Lithium Metal Anode Using Bi-functional Composite Separator: A New Approach for Lithium Battery

Fengquan Liu[1], Tianqi Xiang[2], Jinxin Xue[2], Sixin Jia[2], Jun Yan[2], Hong Huo[2], Jianjun Zhou[2], Lin Li[1,2*]*

[1] College of Textiles & Clothing, State Key Laboratory of Bio-Fibers and Eco-Textiles, Qingdao University, Qingdao, 266071, China

[2] Beijing Key Laboratory of Energy Conversion and Storage Materials, College of Chemistry, Beijing Normal University, Beijing 100875, China

Correspondence and requests for materials should be addressed to J. J. Zhou. (pla_zjj@bnu.edu.cn) and L. Li (lilinll@bnu.edu.cn)

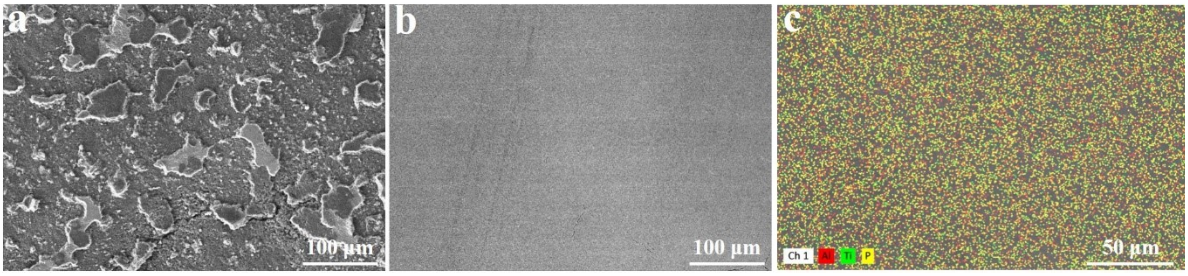

Fig. S1. Top view SEM images of covering layer of 3D Li metal anodes obtained by Li||Cu cells with PP/LATP separator using different binders: (a) PVDF and (b) PEO at 0.5 mA cm^{-2} for 4.0 m Ah cm^{-2} in 1.0 mol L^{-1} LiTFSI electrolyte (DOL:DME =1:1, 1 wt% $LiNO_3$). (c)The element mapping image of covering layer with PEO as binder.

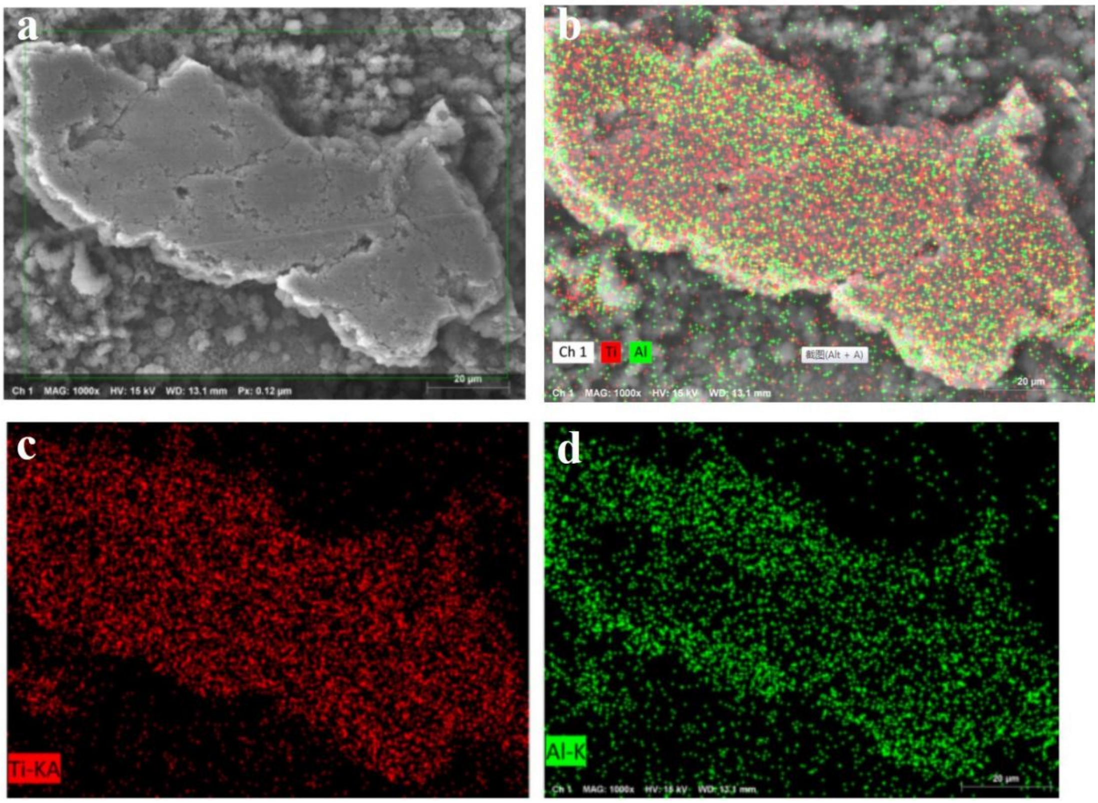

Fig. S2. Top view SEM image and its element mapping images of covering layer of 3D Li metal anode obtained by Li||Cu cell with PP/LATP separator using binder of PVDF at 0.5 mA cm^{-2} for 4.0 mAh cm^{-2} in 1.0 mol L^{-1} LiTFSI electrolyte (DOL:DME =1:1, 1 wt% $LiNO_3$).

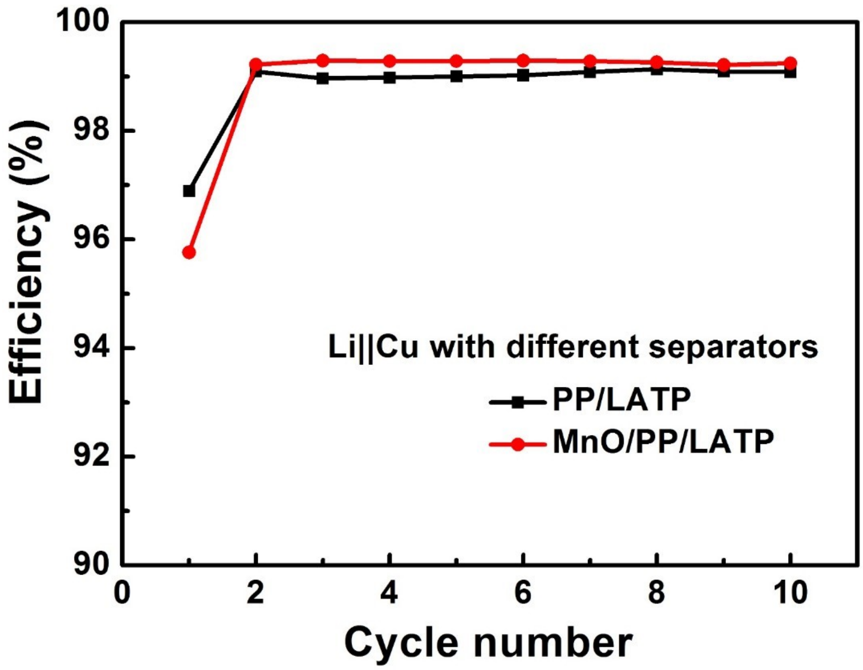

Fig. S3. The Coulombic efficiencies of Li||Cu cells with different separators under 0.5 mA cm^{-2} for 4.0 mAh cm^{-2}, respectively.

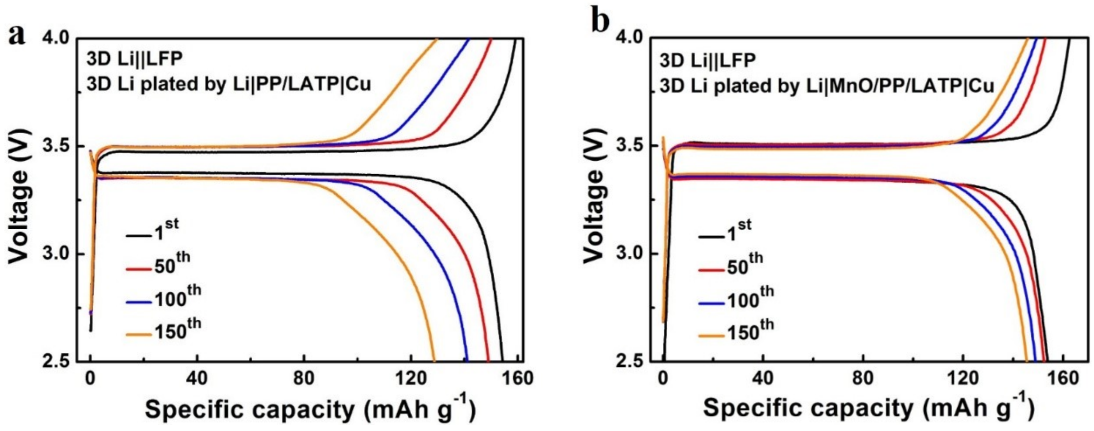

Fig. S4. Charging/discharging curves of 3D Li||LFP cells with PP separators in 1.0 mol L^{-1} LiTFSI (DOL:DME=1:1, 1 wt% $LiNO_3$) at 0.5 C. 3D Li was obtained by Li||Cu cells with different separators in 1.0 mol L^{-1} LiTFSI (DOL: DME=1:1, 1 wt% $LiNO_3$).

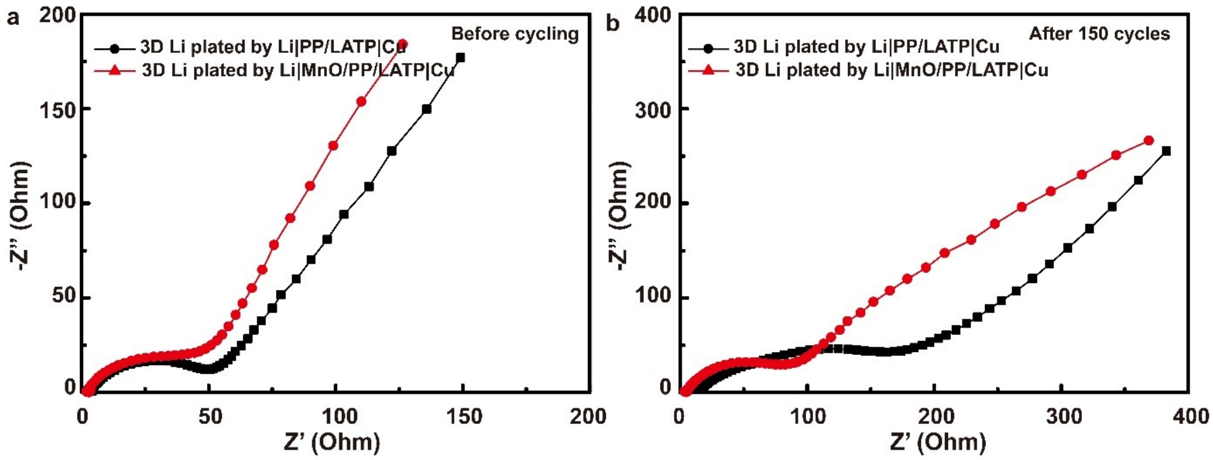

Fig. S5. Nyquist plots of the 3D Li||LFP cells with PP separators before and after 150 cycles.

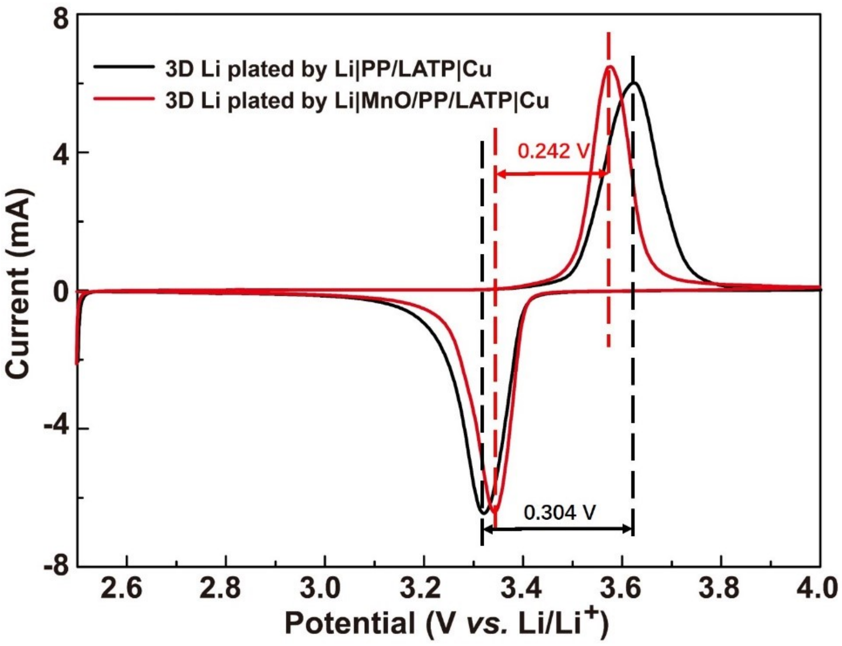

Fig. S6. CV curves of 3D Li||LFP cells at the scan rate of 0.1 mV s^{-1}.

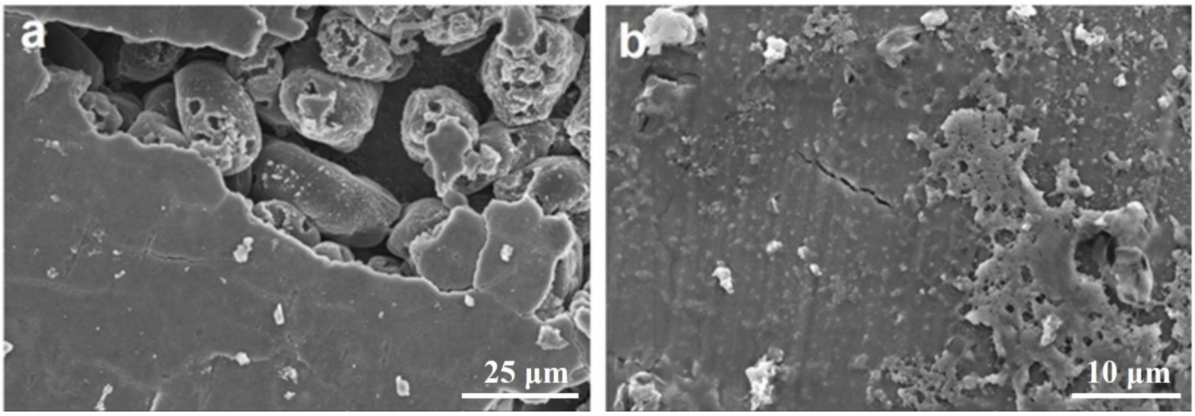

Fig. S7. SEM images of 3D Li anode from 3D Li||NCM622 cells after 80 cycles.

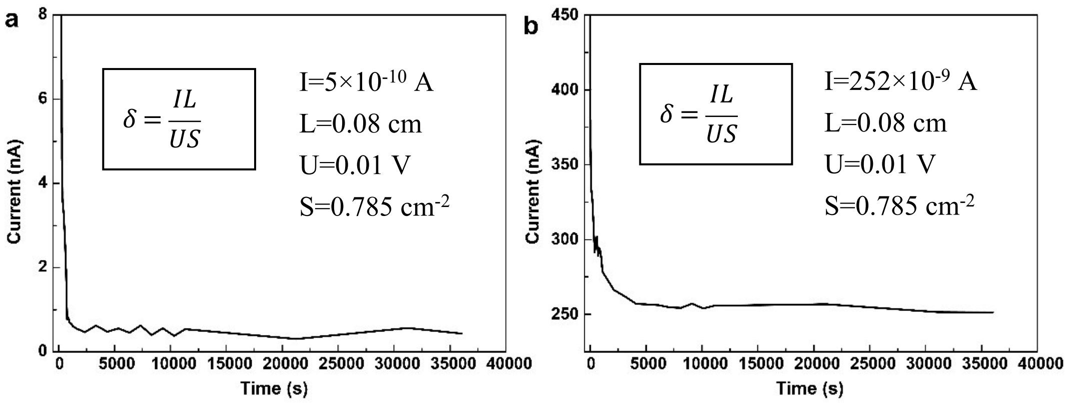

Fig. S8. Curves of direct current with time of pure LATP and reduced LATP.

Table S1. Properties of the separators

	Thickness, μm	Gurly Value, s/100cc	Porosity, %	Puncture Strength, gf	Tensile strength, MPa		Shrinkage, % (120 ^{o}C, 1 h)	
					MD	TD	MD	TD
PP	19.6	257	40.2	378	143.5	15.3	4.5	0
MnO/PP/LATP	27.3	315	39.6	332	136.4	14.2	1.7	0

Table S2. The summary of performance of Li||LFP cells with lithium-free or ultra-thin lithium-metal anodes in the literatures.

Method	Material	Full cells cycling performance					Ref.
		Cathode and areal capacity	Areal capacity of anode	Charge/ discharge rate	Cycling number	Capacity retention rate (%)	
Modifying separator	$MnCO_3$	LFP 3.8 mg cm^{-2}	2.0 mAh cm^{-2}	0.5C/0.5C	110	95.3	[9]
Highly concentrated electrolytes	4.0 M LiFSI-DME	LFP 1.6 mAh cm^{-2}	Anode-free	0.2 mA cm^{-2}/2.0 mA cm^{-2}	100	54	[10]
Coating on Cu current collector	PDMS	LFP	1.0 mAh cm^{-2}	0.5C/0.5C	100	~ 93	[17]
Modifying separator	MnO	LFP 6.0 mg cm^{-2}	2.0 mAh cm^{-2}	0.5C/0.5C	120	98.4	[23]
Modifying Cu current collector	Lithium-copper-lithium arrays	$LiCoO_2$	Li: 70 μm Cu: 25 μm	0.2C/0.2C	200	92	[28]
Modifying Cu current collector	CuO nanoshe-ets	LFP 1.3 mg cm^{-2}	1.5 mAh cm^{-2}	0.5C/0.5C	300	81.3	[29]
Dual-salt carbonate electrolyte	2.0 M LiDFOB and 1.4 M $LiBF_4$	NMC532 3.1 mAh cm^{-2}	Anode-free	0.2C/0.5C	200	80	[30]

Epitaxial induced plating current-collector	Liquid metal coating layer	NCM811 25 mg cm^{-2}	Anode-free	1st: 0.05C/ 0.2C; 2th-50th: 0.1C/0.2C	50	84	[31]
Modifying separator	MnO and LATP	LFP 9.6 mg cm^{-2}	4.0 mAh cm^{-2}	0.5C/0.5C	150	94.5	This work
